# Photoacoustic thermal characterization of low thermal diffusivity thin films

**DOI:** 10.1016/j.pacs.2021.100246

**Published:** 2021-02-26

**Authors:** K. Herrmann, N.W. Pech-May, M. Retsch

**Affiliations:** a Department of Chemistry, Physical Chemistry 1, University of Bayreuth, 95440 Bayreuth, Germany; b Bundesanstalt für Materialforschung und -prüfung (BAM), 12200 Berlin, Germany

**Keywords:** Thermal conductivity, Thermal wave, Thermal transport metrology, Photoacoustic characterization, Effusivity mismatch, Thermal diffusivity, Thin film characterization, Nanoscale thermal transport

## Abstract

The photoacoustic measurement technique is a powerful yet underrepresented method to characterize the thermal transport properties of thin films. For the case of isotropic low thermal diffusivity samples, such as glasses or polymers, we demonstrate a general approach to extract the thermal conductivity with a high degree of significance. We discuss in particular the influence of thermal effusivity, thermal diffusivity, and sample layer thickness on the significance and accuracy of this measurement technique. These fundamental thermal properties guide sample and substrate selection to allow for a feasible thermal transport characterization. Furthermore, our data evaluation allows us to directly extract the thermal conductivity from this transient technique, without separate determination of the volumetric heat capacity, when appropriate boundary conditions are fulfilled. Using silica, poly(methyl methacrylate) (PMMA) thin films, and various substrates (quartz, steel, and silicon), we verify the quantitative correctness of our analytical approach.

## 1 Introduction

Rosencwaig and Gersho proposed the basic theory of the photoacoustic effect in condensed matter in 1976 [1]. A periodically modulated laser beam is guided onto the material. Part or all absorbed light energy is transformed to heat through non-radiative deexcitation processes [2]. Therefore, a periodic heat source is realized. If a solid sample is enclosed in a gas-tight cell, an alternating expansion and contraction of the gas layer adjacent to the solid surface is induced due to the modulated surface temperature [3]. This generates the photoacoustic pressure signal, depending on the properties of the sample [1], [4]. The proposed one-layer model by Rosencwaig and Gersho has been continuously extended to a two- and *N*-layer model to access more complex sample structures [4], [5], [6].

Thermal characterization of thin films is an ongoing research topic since it is highly relevant because in nowadays microelectronics, coatings, or sensors, almost all materials are used as thin films [7], [8], [9]. The main goals are either dissipating the heat away as efficiently as possible, as with computer processors and solar cells, or maintaining a temperature gradient as in thermal barriers or thermoelectrics. There are several high-end thin film characterization methods, like frequency or time-domain thermoreflectance, transient thermal grating, or 3*ω*, which are usually accompanied by a high experimental effort and or revolve around thermally highly conductive materials where the heat carrier mean free path is in the order of the film thickness [10], [11], [12], [13]. Furthermore, specific requirements for the sample geometry and layout cannot always be met by the mentioned techniques. Typical issues are surface roughness, optical transparency, or electrical isolation, respectively. Compared to these techniques, the photoacoustic thermal characterization has been less widely used, which is even more surprising considering its suitability for low thermal diffusivity samples that we want to highlight in this contribution [14].

The photoacoustic thermal characterization technique is comparatively simple and affordable. It can be applied to a wide range of materials with little restriction on the surface roughness, optical properties, or electrical insulation. Based on the existing framework, we develop this technique further to show its feasibility for many low diffusivity materials and the high degree of significance of the data evaluation. The performed analysis is based on the assumption of one-dimensional heat conduction in a multilayer system, in the absence of thermal contact resistances, stating continuity of temperature and heat flux at the interfaces. For ensuring a high signal-to-noise ratio, a closed-cell approach is utilized, where helium can be employed as the gas medium. Furthermore, we focus on the thermal piston effect since the mechanical piston effect can be estimated to contribute less than 1% to the measurement signal [4].

At first, a rigorous sensitivity analysis is performed to outline general dependencies and relationships. Thereby, the influences of thermal effusivity and diffusivity can be understood rather descriptively in the framework of the one-dimensional thermal diffusion equation. Building upon this, uncertainty analysis for the concrete case of a low thermal diffusivity material is performed. In doing so, the limits of the significance of the measurement technique are theoretically covered for such samples.

Experimental data subsequently verify the outlined theoretical framework. Thermally grown SiO_2_ films of 5 μm thickness on silicon are characterized as reference samples. Furthermore, general relationships regarding thermal thickness and effusivity mismatch have been addressed using poly(methyl methacrylate) (PMMA) thin films. For both, a satisfying agreement between measured and literature values is found.

In conclusion, we point out how to optimize and perform the thermal characterization of low diffusivity solids in the μm and sub-μm regime with the in comparison relatively simple experimental setup of the photoacoustic measurement.

## 2 Theory

### 2.1 Thermal wave interferometry

Thermal wave interference is implicitly contained in the Rosencwaig–Gersho theory. Still, it is worth pointing out the role of interference by using an approach incorporating thermal wave reflection and transmission coefficients [15].

Due to the periodic nature of the thermal excitation and therefore a harmonic heat flow, highly damped so-called thermal waves are generated. For thermally thick materials, which are considered semi-infinite, such thermal waves propagate freely into the bulk and exhibit a constant phase shift of the surface temperature relative to the modulated heat source of $\frac{\pi }{2}$ [16]. The surface temperature can hereby directly be related to the photoacoustic signal, as explained in detail in Appendix B.

Introducing a finite layer on top of a semi-infinite one leads to the presence of an interface. This interface prevents the free propagation of thermal waves and results in an altered phase shift Δ*ϕ* relative to the semi-infinite bulk material.

**Fig. 1:**
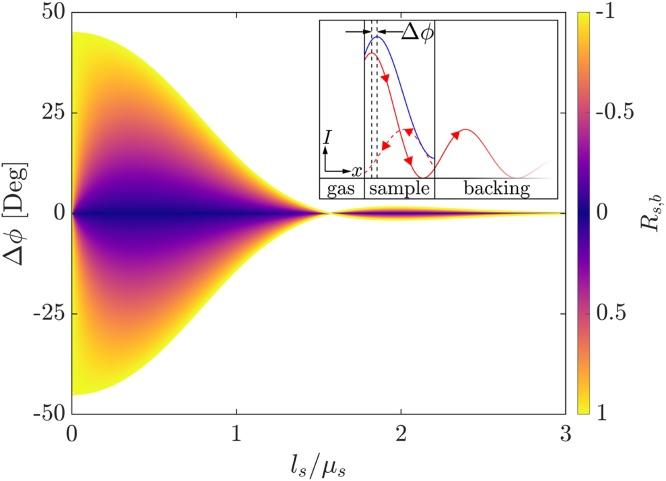
Theoretical phase shift of the surface temperature for a two-layer system with different thermal reflection coefficients *R*_*s*,*b*_ as a function of the frequency-dependent thermal thickness *l*_*s*_/*μ*_*s*_[15]. The inset is intended to depict the principle of thermal wave interference. (For interpretation of the references to color in this figure legend, the reader is referred to the web version of this article.)

The general concept of thermal wave interference can be explained rather simply for a two-layer system with the help of a gedanken experiment. The second layer is considered semi-infinite in this case. A diagram of the gedanken experiment is depicted in the inset of Fig. 1, where *x* denotes the one-dimensional space coordinate, while *I* represents the wave intensity. Here a damped heat wave, comprising diffusive phonon transport, propagates freely in a medium in the positive *x*-direction (solid red line). If wave-like properties were assigned, the introduction of an interface would lead to reflection in the negative *x*-direction (dashed red line), and a superposition within the sample (solid blue line) would be the consequence. This superposition manifests in a phase shift and an altered amplitude, which can be detected by measuring the surface temperature. For illustration purposes, we assumed total reflection at the sample backing interface in Fig. 1. The amount of reflection as well as the thickness, and thus damping, therefore directly influence Δ*ϕ*, which will be further elucidated in the performed sensitivity analysis.

Although the heat transport is diffusive, and, as Salazar pointed out, the thermal waves do not transport energy, wave-like properties such as reflection and interference are still instructive to understand the influence of thermal effusivity mismatch and thermal diffusivity on Δ*ϕ* [17], [18]. When a thermal wave strikes the interface between the sample and backing with thermal effusivities *ε*_*s*_ and *ε*_*b*_ the thermal reflection coefficient *R*_*s*,*b*_ for normal incidence and in the absence of contact resistances reduces to [18]:

(1) $$R_{s,b}=\frac{1-\varepsilon_{b}/\varepsilon_{s}}{1+\varepsilon_{b}/\varepsilon_{s}}=\frac{\varepsilon_{s}-\varepsilon_{b}}{\varepsilon_{s}+\varepsilon_{b}}$$

Therefore, the magnitude of *R*_*s*,*b*_ is determined by the ratio of thermal effusivities, which may be regarded as a measure of the thermal mismatch between the two media [19]. The presence of contact resistances would lead to discontinuities in temperature and heat flux at the interface and are not considered in this work. For samples between the thermally thin and thermally thick limiting cases, thermal wave interference effects are observable and can be utilized for thermal characterization.

In addition to the thermal wave interferometry explanation, fundamental parameters affecting the phase shift on the sample's surface can be identified from the one-dimensional heat diffusion model in a multi-layered system in Appendix A. The phase shift is based primarily on the thermal effusivity ratios *ε*_*i*+1_/*ε*_*i*_ and the dimensionless thermal thickness *l*_*i*_/*μ*_*i*_ (see Eqs. (A.6a) and (A.6b)), and therefore the sample thickness and diffusivity, as well as the modulation frequency of the heat source.

The phase shift can be calculated for different values of *R*_*s*,*b*_ between the sample and the thermally thick backing. By introducing the dimensionless thermal thickness *l*_*s*_/*μ*_*s*_, a representation is obtained, which is independent of the actual values of *D*_*s*_, *l*_*s*_, and the frequency regime is shown in Fig. 1.

For *R*_*s*,*b*_ ≠ 0, a maximum (*R*_*s*,*b*_ < 0, *ε*_*s*_ < *ε*_*b*_) or minimum (*R*_*s*,*b*_ > 0, *ε*_*s*_ > *ε*_*b*_) in phase shift is present. Moreover, the effusivity ratio between sample and backing affects the extremums resulting shape and position. Increasing the absolute value of *R*_*s*,*b*_ thereby shifts the extremum to lower thermal thicknesses (*l*_*s*_/*μ*_*s*_) while the resulting change in phase shift (Δ*ϕ*) also increases in absolute value. For a fixed thermal diffusivity and measurement frequency regime, the sample thickness shifts the extremum's position. Increasing the sample thickness shifts the maximum to lower frequencies, while decreasing it causes a shift to higher frequencies. Considering *R*_*s*,*b*_ < 0, which is a substrate with higher thermal effusivity than the sample, the thermal wave is phase-shifted upon reflection, similar to the behavior of electromagnetic waves. This leads to the symmetric split of the phase shift depending on the sign of *R*_*s*,*b*_. An interesting point is the zero-crossing at *l*_*s*_/*μ*_*s*_ = *π*/2, regardless of the thermal reflection coefficient. At this point, the sample thickness is equal to a quarter of the thermal wave with *λ* = 2*πμ*_*s*_ leading to a zero-crossing. Zero-crossings are theoretical also present for *l*_*s*_/*μ*_*s*_ = *n* · *π*/2 with n∈ℕ, but not recognizable due to the heavily damped nature of the thermal wave.

Summing up, thermal effusivity affects the amount of heat being reflected at the sample backing interface, while thermal diffusivity affects the damping of the thermal wave. In the one-dimensional limit of the heat diffusion equation with a periodic heat flow, general correlations between the effusivity ratios *ε*_*i*+1_/*ε*_*i*_ and thermal thickness *l*_*i*_/*μ*_*i*_, which affect the surface temperature, can now be identified. The question of how these parameters affect the measurement is addressed with the help of sensitivity analysis in Section 2.3.

### 2.2 Model

**Fig. 2:**
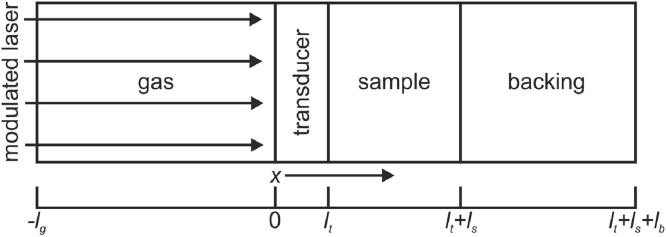
Schematic representation of the modeled sample geometry, including a transducer and a thermally thick backing.

The samples investigated in this work follow a geometry, as shown in Fig. 2. A transducer layer with a high absorption coefficient at the excitation wavelength is used to ensure a sufficiently high photoacoustic signal. Therefore, the sample is exposed to a modulated heat flux instead of heat being generated in the sample itself. This, furthermore, enables the measurement of transparent solids. Additionally, the optical properties of the sample are not relevant for the measurement and data evaluation.

In the following, the subscripts *b*, *s*, *t*, and *g* will represent the backing, sample, transducer, and gas, respectively. For studying thermal transport across an *N*-layered film, Xu et al. proposed a matrix-based solution for the one-dimensional heat diffusion equation [4]. We consider the solution corresponding to three layers (transducer, sample, and backing) in this work. Additionally, heat conduction to the gas is also considered. The mathematical development and physical boundary conditions applied to the one-dimensional heat diffusion equation are given in detail in Appendix A and agree with previous reports [1], [4].

In Eqs. (A6) the main factors affecting the thermal model can be identified. Explicitly those are the effusivity ratios between gas and transducer, transducer and sample, and sample and backing in equations (A.6b) and (A.6c). Furthermore, here, the thermal thicknesses of transducer and sample enter in the exponential terms, while gas and backing are assumed to be thermally thick. Heat generation is taken into account in Eqs. (A.6d) and (A.6e). As the model has low sensitivity to the effusivity ratio between transducer and sample *ε*_*t*_/*ε*_*s*_ in the studied regime, this ratio is expressed in terms of the effusivity ratio between the sample and backing *x* = *ε*_*s*_/*ε*_*b*_ by *ε*_*t*_/*x* · *ε*_*b*_. Due to the high absorption coefficient of the transducer at the wavelength of excitation (1.1 × 10^6^ cm^−1^), the intensity of the pump is decreased by a factor of 1 × 10^−5^ for a 100 nm transducer layer [20]. The model, therefore, gets insensitive to the optical properties of the layers below.

We determine the sample's thermal transport properties via the thermal effusivity ratio between the sample and backing *ε*_*s*_/*ε*_*b*_ and the sample's thermal diffusivity *D*_*s*_, not the thermal conductivity directly. Therefore, specific heat and density, or volumetric heat capacity, are not required as known parameters. Only knowledge about the sample thickness is crucial. In an optimized measurement system, thermal diffusivity and effusivity can both be determined. Hence, sensitivity to both variables is essential to derive the thermal conductivity correctly by [19], [21]:

(2) $$k=\varepsilon \sqrt{D}$$

### 2.3 Sensitivity analysis

A sensitivity analysis was performed to estimate the influence of the sample's thermal properties on the measured phase shift. For this purpose, a local method was applied where the sensitivity to the parameter *i* is defined as [22]:

(3) $$S_{i}=\frac{\partial \phi }{\partial p_{i}}\cdot p_{i}$$

With *p*_*i*_ being the value of parameter *i* and *ϕ* being the phase. The partial derivative is calculated numerically by perturbing the value *p*_*i*_ by 1 % and determining the resulting change in phase. Normalizing by multiplying with the parameter value *p*_*i*_ is performed to compare the sensitivity to properties that are different by orders of magnitude [23].

**Fig. 3:**
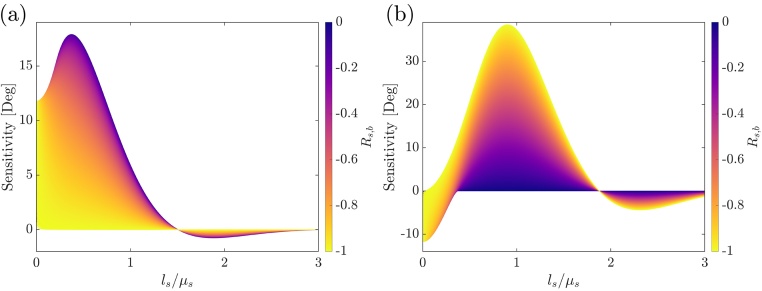
Sensitivity analysis for a polymeric sample to the fit parameters *ε*_*s*_/*ε*_*b*_ (a) and $1/\sqrt{D_{s}}$ (b) as a function of thermal thickness and thermal reflection coefficient.

Only negative reflection coefficients are considered since almost every solid substrate material exhibits a higher thermal effusivity than the polymeric samples investigated in this work. For the sensitivity analysis, the parameters in Table 1 were used. For a polymeric sample with *D*_*s*_ = 0.12 × 10^−6^ m^2^ s^−1^, *ε*_*s*_ = 520 W s^0.5^ m^−2^ K^−1^, a transducer layer of 100 nm nickel and for thermal reflection coefficients −1 ≤ *R*_s,b_ ≤ 0, which represents a variation of the substrate material, the sensitivity analysis is shown in Fig. 3.

**Table 1:** Literature values used for sensitivity and data analysis.^a^

Material	Thermal effusivity [W s^0.5^ m^−2^ K^−1^]	Thermal diffusivity [m^2^ s^−1^]
Helium	17.4^b^	Not needed
Nickel	18932	22.95 × 10^−6^
Quartz	1503	Not needed
Steel AISI 316	7188	Not needed
Silicon	15669	Not needed

a Values are taken from Ref. [18] if not stated explicitly.

b Value is taken from Ref. [26].

An interesting point is the inversed dependency of the two fit parameters on the thermal reflection coefficient *R*_*s*,*b*_. While the sensitivity to the effusivity ratio *ε*_*s*_/*ε*_*b*_ is maximized for a thermal reflection coefficient of zero (see Fig. 3a), meaning the sample and the substrate exhibit the same thermal effusivity, the sensitivity to the thermal thickness is non-existing in this case (see Fig. 3b). With increasing the thermal mismatch, and therefore *R*_*s*,*b*_ approaching −1, the sensitivity to the effusivity ratio decreases. On the other hand, the sensitivity to the thermal diffusivity increases with an increased thermal mismatch.

Briefly, for estimating both thermal effusivity and diffusivity, the thermal reflection coefficient *R*_*s*,*b*_ is supposed to be in an intermediate range, which means sample and substrate have thermal effusivities in the same order of magnitude, while a thermal thickness regime between approximately 0.1 to 1 thermal thicknesses *l*_*s*_/*μ*_*s*_ should be covered.

### 2.4 Uncertainty analysis

**Fig. 4:**
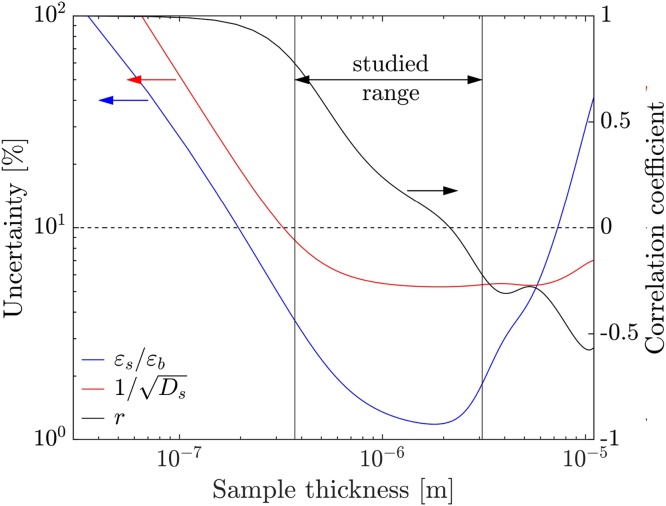
Calculated uncertainties and correlation coefficient *r* of the fit parameters for a polymeric thin film on quartz, plotted as a function of sample thickness. The dotted line is a guide to the eye for 10 % uncertainty and no correlation, while the range of sample thicknesses studied in this work is highlighted.

To determine in which thickness regime samples can be measured significantly, besides the general dependencies covered in the sensitivity analysis, an uncertainty analysis was carried out. Yang et al. developed a procedure to take into account uncertainties in so-called controlled parameters [24]. The general idea is to treat all errors as Gaussian distributed and to approximate the non-linear model in close proximity to the determined fit parameters as a first-order Taylor expansion. This approach was furthermore validated using Monte Carlo simulations [24]. The procedure to calculate the uncertainties is shown in Appendix C. A fixed frequency regime between 310 Hz and 10 kHz is used to relate this uncertainty analysis to the measurement. The uncertainties in the controlled parameters transducer and sample thickness are estimated to be 5 %, and the uncertainty of the measured phase values are taken to be 0.5 °. All other parameters are the same as in the sensitivity analysis except for a fixed thermal reflection coefficient of −0.48, which is approximated for a polymeric sample on quartz and supposed to offer sensitivity to thermal effusivity and diffusivity, based on the sensitivity analysis in Fig. 3. The uncertainty of the thermal properties is taken as the ±1/*e* confidence interval in this paper. The calculated uncertainties and the correlation coefficient *r*, a measure of the strength and direction of the relationship between two variables, for the two fit parameters are shown in Fig. 4.

In general, for thinner and thicker samples than studied in this work, an approximately exponential increase in uncertainty is observable. The uncertainty for the thermal effusivity is lower than the uncertainty for the thermal diffusivity for sample thicknesses below and vice versa above 6 μm. This is due to the different positions of maximum sensitivity in thermal thickness, where the maximum sensitivity for thermal effusivity is present at lower thermal thicknesses than the maximum sensitivity for thermal diffusivity. Furthermore, for low sample thicknesses, the parameters get highly correlated with *r* approaching unity. This renders a simultaneous determination impossible due to the existence of multiple solutions. An optimum sample thickness regime for a polymeric sample can be identified, where both estimated uncertainties are below 10 %. This is the case for sample thicknesses between approximately 500 nm and 5 μm, which can hardly be analyzed by other measurement techniques.

Therefore, the photoacoustic measurement is inherently well suited for thermally characterizing low thermal diffusivity thin films with a high degree of significance. To confirm these numerical calculations, various thin films were examined to estimate how they could be translated into actual measurements.

## 3 Experiment

### 3.1 Samples

Silicon wafers (n-type) with a 5 μm thermally grown SiO_2_ layer were purchased from MicroChemicals. Polymeric samples were prepared using the spin-coating technique. Quartz substrates were bought from Präzisions Glas und Optik GmbH, steel AISI 316 substrates were bought from Goodfellow GmbH, while undoped silicon (111) substrates were purchased from MicroChemicals GmbH. Prior to spin-coating, the substrates were cleaned using ultrasonication in a detergent solution (2 V% Hellmanex III in Milli-Q water) twice and in ethanol p.a. once.

For spin-coating various concentrations of poly(methyl methacrylate) (PMMA) 7N, purchased from Evonik Industries, in chlorobenzene were prepared.

All samples and a thermally thick reference material (quartz) to determine the setup's transfer function were coated with a 100 nm nickel layer by thermal evaporation [16]. The layer thickness was monitored using a quartz crystal microbalance and verified with AFM measurements.

The thicknesses of the polymeric films were determined using an Olympus OLS5000 laser confocal microscope.

### 3.2 Photoacoustic measurement

**Fig. 5:**
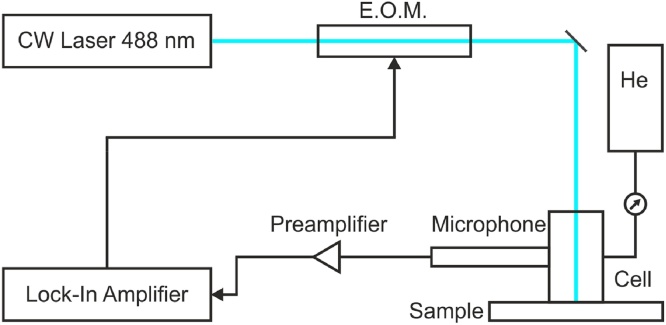
Schematic setup with a continuous wave laser being passed through an EOM to provide the modulated heat source. The photoacoustic signal is measured using a microphone in the pressurized cell.

Photoacoustic measurements were performed with a continuous wave Coherent Genesis MX488-1000 laser. The laser is modulated using a ConOptics 350-160 electro-optic modulator driven by a ConOptics M25A amplifier and a sine signal of a Zurich Instruments lock-in amplifier HF2LI. The generated acoustic signal was detected using a Bruel & Kjaer 4398-A-011 microphone. The signal was then amplified with a Bruel & Kjaer 2690-0S1 preamplifier by 1 V/Pa. The settling time after changing a sweep parameter, as well as the averaging time, was set to 30 s. The laser power was measured to be 35 mW at the sample position using a Coherent FieldMaxII. The 1/*e* diameter of the spot was determined to be 2.19 mm using a DataRay Beam’R2 XY Scanning Slit Beam Profiler. The pressure in the photoacoustic cell was set to 1.379 bar of helium, which corresponds to 20 psi. Helium was used as the cell gas for this work because of its high thermal conductivity, leading to a high signal to noise ratio. The experimental setup is shown schematically in Fig. 5.

The cell itself is made of MACOR® to prevent cell fracture and consists of a cell volume of 4 mm in diameter, which corresponds to the measured sample area, and 8.4 mm height. The microphone is connected via a side bore of 2.2 mm in diameter, and the helium connection via a side bore of 1.6 mm in diameter [22], [25]. Sealing of the samples at the backside of the cell is realized using a ring seal. The front side of the cell is sealed with a sapphire window using epoxy resin.

The true phase shift of the sample, without the setup's transfer function, is calculated as Δ*ϕ* = *ϕ*_sample_ − *ϕ*_reference_, where *ϕ*_sample_ is the measured phase shift for the respective sample and *ϕ*_reference_ is the measured phase shift of a thermally thick quartz sample [22].

### 3.3 Data analysis

Appendix A describes the model being used to perform data analysis. A least-squares fitting method employing the Levenberg–Marquardt algorithm is performed to determine the parameters *ε*_*s*_/*ε*_*b*_ and $1/\sqrt{D_{s}}$. The approach for error estimation is described in Appendix D. In doing so, three independent measurements are analyzed using a Monte Carlo approach for the controlled parameters. Simultaneously, the uncertainty of every fit procedure is taken into account by the respective covariance matrix.

The literature values used for the data analysis are shown in Table 1.

## 4 Results and discussion

In contrast to previous works, we consider the need and influence of an optimized measurement for performing data analysis with a high degree of significance. In doing so, we verify our theoretical findings from Section 2 on a silicon dioxide sample reference with known properties. Subsequently, the proposed approach is applied to polymeric thin films to extract their thermal effusivity and diffusivity simultaneously.

**Fig. 6:**
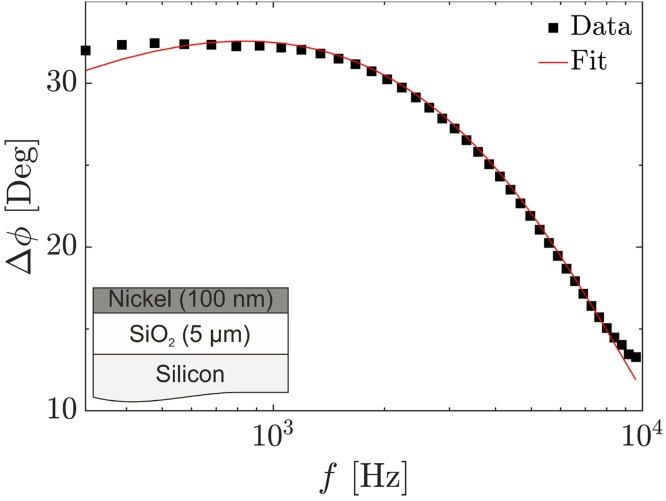
Phase shift data of a 5 μm thermally grown SiO_2_ on a Si substrate with an exemplary performed fit.

As with any measurement technique, testing a sample with known properties is crucial to ensure the accuracy and reproducibility of the performed measurements. Silicon with a thermally grown silicon dioxide layer is one of the most commonly used calibration samples for the photoacoustic technique to validate the present setup before moving on to self-produced polymeric thin films [23]. A 525 μm thick silicon wafer with a 5 μm thick silicon dioxide layer is appropriate for this purpose since it is on the lower boundary of the significantly analyzable sample thickness compared to its inherent thermal diffusivity. This is an improvement over previous work, as they use reference samples with insufficient layer thickness, hence suboptimal sensitivity. The phase shift data of this silicon dioxide on silicon sample with an exemplary performed fit is depicted in Fig. 6.

The extracted thermophysical properties, as well as literature values, are listed in Table 2. A good agreement between the literature and determined thermal properties is evident, verifying this technique's use.

**Table 2:** Extracted thermophysical properties in comparison to literature values. Thermal conductivity is calculated based on thermal diffusivity and effusivity.

Material	Substrate	Thermal diffusivity *D* [×10^−6^ m^2^ s^−1^]	Thermal effusivity *ε* [W s^0.5^ m^−2^ K^−1^]	Thermal conductivity *k* [W m^−1^ K^−1^]
Silicon dioxide				
Literature values^a^		0.87	1503	1.4
*l* = 5 μm	Silicon	0.809 ± 0.013	1560 ± 49	1.40 ± 0.05
PMMA				
Literature values		0.12^b^	522^c^	0.18
* l* = 370 ± 19 nm	Quartz	0.146 ± 0.024	490 ± 26	0.188 ± 0.019
*l* = 1095 ± 55 nm	Quartz	0.119 ± 0.013	507 ± 8	0.175 ± 0.010
*l* = 3096 ± 155 nm	Quartz	0.112 ± 0.012	540 ± 10	0.181 ± 0.010
*l* = 1120 ± 56 nm	Steel	0.117 ± 0.012	552 ± 11	0.189 ± 0.011
* l* = 1075 ± 54 nm	Silicon	0.111 ± 0.012	509 ± 26	0.169 ± 0.013

a Values are taken from Ref. [18].

b Value is taken from Ref. [27].

c Value is taken from Ref. [28].

To demonstrate the crucial influence of the thermal reflection coefficient and the thermal thickness of the samples to be measured, a series of poly(methyl methacrylate) thin films is investigated where the thickness and substrate material, and therefore effusivity mismatch, are varied. Poly(methyl methacrylate) was chosen as a well-characterized, basic polymeric material to verify general dependencies. As substrates materials, silicon, steel AISI 316, and quartz were used to cover a wide range of thermal effusivities.

**Fig. 7:**
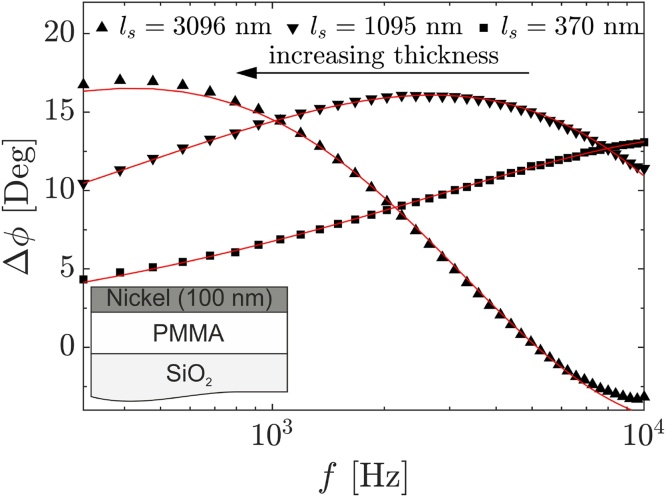
Phase shift data of PMMA thin films on quartz substrates. Exemplary performed fits are depicted by red lines in each case.

The influence of the thermal thickness is investigated on PMMA thin films with thicknesses of 370 ± 19, 1095 ± 55, and 3096 ± 155 nm, respectively, on quartz substrates. The measured phase shift and exemplary performed fits are shown in Fig. 7.

The thickness dependency could be presented convincingly, and quantitative data analysis with a high degree of significance is possible. It is clearly recognizable that the maximum in phase is shifted to lower frequencies with increasing sample thickness. The slight increase of the maximums absolute value in phase shift Δ*ϕ* with increasing layer thickness can, furthermore, be attributed to the presence of a transducer layer. For opaque samples without a transducer layer, the maximums absolute value would be thickness-independent.

Substantial deviations from the sample thicknesses shown here (<500 nm or >3 μm) lead to a shift of the maximum outside of the detectable frequency regime. Hence, only a monotonous increase or decrease in phase shift is detected, making a significant data analysis very difficult.

Compared to literature values, the determined thermal properties in Table 2 based on the input parameters in Table 1 are reasonable. For the thinnest film, deviations start to become apparent due to the increasing uncertainty, as well as the increasing correlation of the parameters to be determined.

**Fig. 8:**
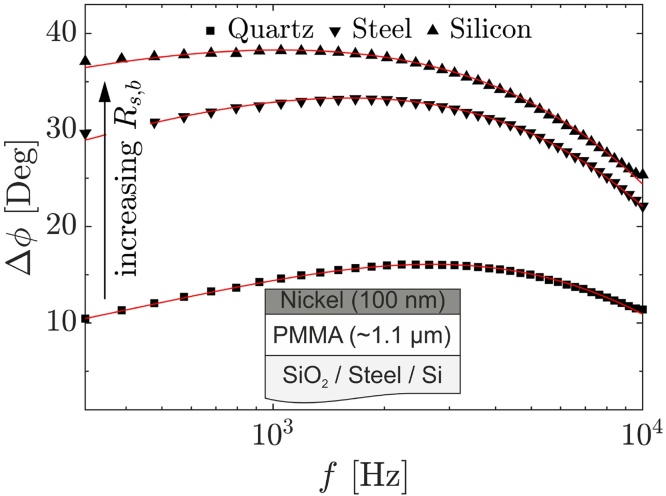
Phase shift data of approximately 1.1 μm PMMA thin films on various substrates, for precise thickness see Table 2. Exemplary performed fits are depicted by red lines in each case.

The influence of thermal effusivity mismatch between sample and substrate is investigated on three different substrate materials. PMMA thin films with thicknesses of 1095 ± 55 nm on quartz, 1120 ± 56 nm on steel AISI 316, and 1075 ± 54 nm on silicon are studied for this purpose. The measured phase shift and exemplary performed fits are shown in Fig. 8.

The influence of effusivity mismatch could likewise be clearly confirmed. Additionally, the determined thermal properties show an evident consistency with the literature values. While a high sensitivity to thermal diffusivity was expected due to the sample thickness for all substrates, the thermal effusivity could still be determined quite accurately for all substrates. Still, the uncertainty is increased on the silicon substrate representing the decreasing sensitivity at a high effusivity mismatch.

These experiments confirm that the photoacoustic measurement technique enables the full thermal characterization of low thermal diffusivity materials in the micron and submicron regime. General dependencies affecting the measurement and how to efficiently design the sample environment have been pointed out. These are crucial to enable highly sensitive measurements of both thermal diffusivity and effusivity.

Furthermore, the employed analytical approach can be applied to other samples and other measurement techniques working with the one-dimensional approximation of the heat diffusion equation.

## 5 Conclusion

In this work, we introduced a reliable approach to extract the thermal conductivity of low thermal diffusivity thin films using the photoacoustic technique. In particular, thermal conductivity uncertainties of around 6–10 % were obtained for PMMA thin films with thicknesses on the order of microns and a few hundreds of nanometers, respectively. Furthermore, we discussed the influence of parameters such as thermal effusivity, thermal diffusivity, and sample layer thickness on the significance and accuracy of this measurement technique. This was performed within the framework of a one-dimensional heat conduction model in a three-layer system. From this analysis, we deduced guidelines for the selection of sample and substrate configuration allowing for a feasible thermal transport characterization of any low thermal diffusivity thin film. Possible applications are, therefore, the accurate thermal characterization of polymeric thin films or emerging hybrid thermoelectric materials. Moreover, our results show that the developed data evaluation allows to directly extract the thermal conductivity from a single measurement, provided that the appropriate boundary conditions are fulfilled.

## Conflict of interest

None declared.

## Declaration of Competing Interest

The authors report no declarations of interest.

## Appendix A Multilayer model

The model from Hu et al. can be adapted for the *N* = 2 layer system depicted in Fig. 2 with a highly absorbing transducer layer [4]. The backing layer is thereby represented as *i* = 0, the sample as *i* = 1, the transducer as *i* = 2, and the gas as *i* = 3. Neither the gas nor the backing is assumed to absorb any of the incident radiation and are considered to be thermally thick. Furthermore, multiple reflections between interfaces and convective heat transfer in the gas are neglected. Thermal contact resistances are not taken into account in this work, and one-dimensional heat diffusion is assumed. For easy comparison with the Rosencwaig–Gersho model, the same notation is adopted. Therefore *f* or *ω* = 2*πf* is the modulation frequency, *β*_*i*_ the optical absorption coefficient, *k*_*i*_ the thermal conductivity of material *i*, *ε*_*i*_ the thermal effusivity of material *i*, *D*_*i*_ the thermal diffusivity of material *i*, $a_{i}=\sqrt{\pi f/D_{i}}$ the thermal diffusion coefficient of material *i*, *σ*_*i*_ = (1 + *j*)*a*_*i*_ the complex-valued thermal diffusion coefficient of material *i* and *θ*_*i*_ = *T*_*i*_ − *T*_amb_ represents the temperature rise of material *i* with respect to the ambient temperature.

In general, at low frequencies (2*πfτ* ≪ 1), where *τ* is the thermal relaxation time, both parabolic and hyperbolic solutions coincide, which is expected to be the case in the frequency regime below 20 kHz.

It has been recognized that the one-dimensional heat transfer model is adequate for describing the temperature variation under typical experimental conditions when the thermal diffusion length in gas and the target is much less than the diameter of the laser beam [29]. Therefore, the thermal diffusion equation in layer *i* can then be expressed as [16]:

(A.1) $$\frac{\partial \theta_{i}}{\partial x^{2}}=\frac{1}{D_{i}}\frac{\partial \theta_{i}}{\partial t}-\frac{\beta_{i}I_{0}}{2k_{i}}\exp(-\sum_{m=i+1}^{N}-\beta_{m}l_{m})e^{\beta_{i}(x-l_{i})}(1+e^{j\omega t})$$

Here *θ*_*i*_ = *T*_*i*_ − *T*_amb_ is the modified temperature in layer *i*. The solution *θ*_*i*_ in general consists of three parts: a transient component, a steady direct component, and a steady alternating component. Since the experiment uses lock-in detection, only the steady alternating component ${\tilde{\theta }}_{i}$ is measured, only this component needs to be evaluated [16]. It results from the periodic source term in Eq. (A.1) and has a particular solution in the form of −*E*_*i*_*e*^*β*_i_(*x*−*l*_i_)^*e*^*jωt*^, where $E_{i}=G_{i}/(\beta_{i}^{2}-\sigma_{i}^{2})$ with $G_{i}=(\beta_{i}I_{0})/(2k_{i})\exp(-\sum_{m=i+1}^{N}\beta_{m}l_{m})$ for *i* < *N*, *G*_*N*_ = *β*_*N*_*I*_0_/2*k*_*N*_, and *G*_*N*+1_ = 0 [16]. The general solution can be expressed in the form of:

(A.2) $${\tilde{\theta }}_{i}=[A_{i}e^{\sigma_{i}(x-h_{i})}+B_{i}e^{-\sigma_{i}(x-h_{i})}-E_{i}e^{x-h_{i}}]e^{j\omega t}$$

*A*_*i*_ and *B*_*i*_ are coefficients to be determined, while *h*_*i*_ is calculated as *h*_*i*_ = *l*_*i*_ for *i* < *N* and *h*_*N*+1_ = 0. To solve this, the gas and backing layer are assumed to be thermally thick, meaning |*σ*_0_*l*_0_| ≫ 1 and |*σ*_*g*_*l*_*g*_| ≫ 1. Based on this assumption, the coefficients *A*_*N*+1_ and *B*_0_ can be taken as zero. Using interfacial conditions, stating that the heat flux is continuous between layers,

(A.3) $$k_{i}\frac{\partial {\tilde{\theta }}_{i}(l_{i})}{\partial x}-k_{i+1}\frac{\partial {\tilde{\theta }}_{i+1}(l_{i})}{\partial x}=0$$

Similarly, continuity of the temperature at the interfaces is assumed,

(A.4) $${\tilde{\theta }}_{i}(l_{i})-{\tilde{\theta }}_{i+1}(l_{i})=0$$

the recurrence formula of the coefficients *A*_*i*_ and *B*_*i*_ is obtained in matrix form:

(A.5) $$\left[\begin{array}{c} A_{i}\\ B_{i} \end{array}\right]=U_{i}\left[\begin{array}{c} A_{i+1}\\ B_{i+1} \end{array}\right]+V_{i}\left[\begin{array}{c} E_{i}\\ E_{i+1} \end{array}\right]$$

where

(A.6a) $$U_{i}=\frac{1}{2}\left[\begin{array}{cc} u_{11,i} & u_{12,i}\\ u_{21,i} & u_{22,i} \end{array}\right];\;V_{i}=\frac{1}{2}\left[\begin{array}{cc} v_{11,i} & v_{12,i}\\ v_{21,i} & v_{22,i} \end{array}\right]$$

(A.6b) $$u_{1n,i}=\left(1\pm \frac{\varepsilon_{i+1}}{\varepsilon_{i}}\right)\cdot \exp\left(\mp (1+j)\frac{l_{i+1}\cdot \sqrt{\pi f}}{\sqrt{D_{i+1}}}\right)$$

(A.6c) $$u_{2n,i}=\left(1\mp \frac{\varepsilon_{i+1}}{\varepsilon_{i}}\right)\cdot \exp\left(\mp (1+j)\frac{l_{i+1}\cdot \sqrt{\pi f}}{\sqrt{D_{i+1}}}\right)$$

(A.6d) $$v_{n1,i}=1\pm \frac{\beta_{i}}{\sigma_{i}}$$

(A.6e) $$v_{n2,i}=\left(-1\mp \frac{k_{i+1}\beta_{i+1}}{\varepsilon_{i}(1+j)\sqrt{\pi f}}\right)\cdot \exp(-\beta_{i+1}l_{i+1})$$

The coefficients *A*_*i*_ and *B*_*i*_ are obtained as:

(A.7) $$B_{N+1}=-\frac{\left[\begin{array}{cc} 0 & 1 \end{array}\right]\sum_{m=0}^{N}\left(\prod_{i=0}^{m-1}U_{i}\right)V_{m}\left[\begin{array}{c} E_{m}\\ E_{m+1} \end{array}\right]}{\left[\begin{array}{cc} 0 & 1 \end{array}\right]\left(\prod_{i=0}^{N}U_{i}\right)\left[\begin{array}{c} 0\\ 1 \end{array}\right]}$$

(A.8) $$\left[\begin{array}{c} A_{i}\\ B_{i} \end{array}\right]=\left(\prod_{m=i}^{N}U_{m}\right)\left[\begin{array}{c} 0\\ B_{N+1} \end{array}\right]+\sum_{m=i}^{N}\left(\prod_{k=i}^{m-1}U_{k}\right)V_{m}\left[\begin{array}{c} E_{m}\\ E_{m+1} \end{array}\right]$$

where $\prod_{k=i}^{i-1}U_{k}$ is taken as $\left[\begin{array}{cc} 1 & 0\\ 0 & 1 \end{array}\right]$. In particular, the complex time-dependent temperature distribution in the gas can be found as [16]:

(A.9) $$\theta_{N+1}=B_{N+1}\cdot \exp\left(-\sigma_{N+1}x\right)\exp\left(j\omega t\right)$$

## Appendix B Photoacoustic signal generation

The measured photoacoustic pressure signal is due to the acoustic wave in the gas cell induced by the incident light on the sample [4]. To draw conclusions about the temperature oscillations at the sample surface by evaluating the generated acoustic signal, surface temperature and pressure have to be set into a relation. According to the basic principle of thermodynamics, there are relations among intensive thermodynamic properties, namely the equations of state [4]. For the gas in the photoacoustic cell, the pressure can, therefore, be expressed by temperature and volume. Assuming an ideal gas behavior yields the shown simplification:

(B.1) $$dp={\left(\frac{\partial p}{\partial T}\right)}_{V}dT+{\left(\frac{\partial p}{\partial V}\right)}_{T}dV=\frac{p}{T}dT-\frac{p}{V}dV$$

As the dimension of the cell *l*_*g*_ is required to be less than half of the minimum wavelength of the sound wave in the gas cell during the measurement, the pressure can be assumed uniform [4]. As there is a temperature distribution due to the incident radiation, the differential temperature *dT* in Eq. (B.1) can be replaced by a volume-averaged temperature variation 〈*dT*〉 yielding

(B.2) $$dp=\frac{p}{T}\langle dT\rangle -\frac{p}{V}dV$$

The differential pressure *dp* generates the photoacoustic signal. As shown by Hu et al., this can be interpreted as a composite piston model with the differential temperature being related to a thermal piston, while the differential volume is related to a mechanical piston [4]. We focus on the thermal piston effect since the mechanical piston effect can be estimated to contribute less than 1 % to the measurement signal. As the volume average of the temperature 〈*dT*〉 in Eq. (B.2) is equivalent to the modified temperature in the gas due to periodic heating of the sample 〈*θ*_*N*+1_〉 as derived in Eq. (A.9), the measured photoacoustic signal phase can be reduced to the surface temperature phase $\mathrm{Arg}(B_{N+1})-\frac{\pi }{4}$ [4].

This relationship is used to determine the unknown parameters in *B*_*N*+1_ by fitting the theoretical model to the experimentally measured phase-shift data. If sample thickness and transducer and backing properties are known, the sample's thermal properties can be extracted.

The difference between the piston models developed by Hu *et al.* compared to the ones developed by Rosencwaig and Gersho lies in the ratio of specific heats $\gamma =c_{p}/c_{v}$, which is apparent in the RG theory [1], [4]. In the model by Rosencwaig and Gersho, both the thermal and mechanical compressing processes in the gas cell are assumed to be adiabatic. However, the process is isochoric for the thermal piston and polytropic for the mechanical piston as shown by Hu et al [4].

## Appendix C Uncertainty analysis

Uncertainty analysis was carried out according to Yang et al. [24]. Thereby, the measurement uncertainties of the phase signal and uncertainties in the controlled parameter are taken into account. The controlled parameters contributing the largest uncertainties in this measurement are the transducer and sample thickness. Thermal properties of backing, transducer, and gas materials are taken as absolute values. Jacobian matrices for the unknown and controlled parameters were calculated numerically by a local approach of perturbing the parameter of interest and evaluating the resulting change in phase. By assuming that the controlled parameters, as well as the experimental noise, are Gaussian distributed around their mean values and that both are independent variables, the variance-covariance matrix of the unknown parameters is given by [24]:

(C.1) $$\begin{array}{cc} \mathrm{Var}\left[{\hat{X}}_{U}\right] & ={\left({J_{U}^{★}}^{\prime }J_{U}^{★}\right)}^{-1}{J_{U}^{★}}^{\prime }(\mathrm{Var}[\Phi ]\\ & \;+J_{C}^{★}\mathrm{Var}\left[X_{C}\right]{J_{C}^{★}}^{\prime })J_{U}^{★}{\left({J_{U}^{★}}^{\prime }J_{U}^{★}\right)}^{-1} \end{array}$$

with the Jacobians for unknown ($J_{U}^{★}$) and controlled parameters ($J_{C}^{★}$)

(C.2a) $$J_{U}^{★}=\left(\begin{array}{cc} \frac{\partial \phi (f_{1},X_{U},X_{C})}{\partial x_{1}}{|}_{X_{U}^{★},X_{C}^{★}} & \frac{\partial \phi (f_{1},X_{U},X_{C})}{\partial x_{2}}{|}_{X_{U}^{★},X_{C}^{★}}\\ \vdots & \vdots \\ \frac{\partial \phi (f_{M},X_{U},X_{C})}{\partial x_{1}}{|}_{X_{U}^{★},X_{C}^{★}} & \frac{\partial \phi (f_{M},X_{U},X_{C})}{\partial x_{2}}{|}_{X_{U}^{★},X_{C}^{★}} \end{array}\right)$$

(C.2b) $$J_{C}^{★}=\left(\begin{array}{cc} \frac{\partial \phi (f_{1},X_{U},X_{C})}{\partial x_{3}}{|}_{X_{U}^{★},X_{C}^{★}} & \frac{\partial \phi (f_{1},X_{U},X_{C})}{\partial x_{4}}{|}_{X_{U}^{★},X_{C}^{★}}\\ \vdots & \vdots \\ \frac{\partial \phi (f_{M},X_{U},X_{C})}{\partial x_{3}}{|}_{X_{U}^{★},X_{C}^{★}} & \frac{\partial \phi (f_{M},X_{U},X_{C})}{\partial x_{4}}{|}_{X_{U}^{★},X_{C}^{★}} \end{array}\right)$$

where $X_{U}^{★}$ is a column vector with the least-square estimates of the unknown parameters, $X_{C}^{★}$ is a column vector with the determined controlled parameters, *M* is the number of measurement points, *x*_1_ and *x*_2_ are the unknown fit parameters *ε*_*s*_/*ε*_*b*_ and $1/\sqrt{D_{s}}$, *x*_3_, and *x*_4_ the controlled parameters *l*_*t*_ and *l*_*s*_, respectively. Furthermore, ${J_{U}^{★}}^{\prime }$ represents the transpose of $J_{U}^{★}$. The variance-covariance matrix of the measured phase and the controlled parameters can be written as

(C.3a) $$\mathrm{Var}\left[\Phi \right]=\left(\begin{array}{cccc} \sigma_{\phi_{1}}^{2} & 0 & \cdots & 0\\ 0 & \sigma_{\phi_{2}}^{2} & \cdots & 0\\ \vdots & \vdots & \ddots & 0\\ 0 & 0 & 0 & \sigma_{\phi_{M}}^{2} \end{array}\right)$$

(C.3b) $$\mathrm{Var}\left[X_{C}\right]=\left(\begin{array}{cc} \sigma_{l_{t}}^{2} & 0\\ 0 & \sigma_{l_{s}}^{2} \end{array}\right)$$

where $\sigma_{\phi_{i}}^{2}$ is the variance of the measured phase data point at the modulation frequency *f*_*i*_ and $\sigma_{l_{t}}^{2}$ and $\sigma_{l_{s}}^{2}$ are the variances of the controlled parameters.

The variances of the unknown parameters can then be retrieved from the diagonal elements of Eq. (C.1) using the experimentally determined variances in the phase data and the variances in the controlled parameters:

(C.4) $$\mathrm{Var}\left[{\hat{X}}_{U}\right]=\left(\begin{array}{cc} \sigma_{x_{1}}^{2} & \mathrm{cov}\left[x_{1},x_{2}\right]\\ \mathrm{cov}\left[x_{1},x_{2}\right] & \sigma_{x_{2}}^{2} \end{array}\right)$$

Therefore, the standard deviation of the determined parameters is given by the square root of the diagonal elements, while the off-diagonal elements represent the covariance between the two parameters. The correlation coefficient is determined as $r=\frac{\mathrm{cov}\left[x_{1},x_{2}\right]}{\sigma_{x_{1}}\cdot \sigma_{x_{2}}}$.

## Appendix D Error estimation

Error estimation on the determined thermophysical properties was done by joining Monte Carlo error estimations with Jacobian based confidence intervals. As in Appendix C, the controlled parameters for the Monte Carlo error estimation are transducer and sample thickness. Based on the assumption of Gaussian distributed errors in the controlled parameters, the Monte Carlo error estimation was carried out. Therefore, 1000 iterations with randomly selected controlled parameters on three data sets, measured on the same sample but at different positions, were performed using the *nlinfit* algorithm in Matlab. For every iteration, the confidence intervals, based on the Jacobian and the mean square error resulting from the non-linear regression algorithm of the fit parameters were collected using *nlparci* in Matlab. Afterward, the errors due to uncertainty in the controlled parameters and the errors due to fitting, resulting from the Jacobian matrices and the mean square error, were added following $\frac{\Delta x}{x}=\sqrt{{\left(\frac{\Delta x}{x}\right)}_{\mathrm{Monte}\mathrm{Carlo}}^{2}+{\left(\frac{\Delta x}{x}\right)}_{\mathrm{Fit}}^{2}}$. Still, all errors were assumed to be Gaussian distributed, and the ±1/*e* confidence intervals are used as error bars.

The resulting error in thermal conductivity was then calculated using [30]:

(D.1) $$\sigma_{k}^{2}={\left(\frac{\partial k}{\partial \varepsilon }\right)}^{2}\sigma_{\varepsilon }^{2}+{\left(\frac{\partial k}{\partial D}\right)}^{2}\sigma_{D}^{2}+2\frac{\partial k}{\partial \varepsilon }\frac{\partial k}{\partial D}\mathrm{cov}\left[\varepsilon ,D\right]$$

Substituting Eq. (2) into Eq. (D.1) yields

(D.2) $$\sigma_{k}^{2}=D\sigma_{\varepsilon }^{2}+\frac{\varepsilon^{2}}{4D}\sigma_{D}^{2}+\varepsilon \cdot \mathrm{cov}\left[\varepsilon ,D\right]$$

where the average covariance regarding the performed Monte Carlo iterations and different measurements was used.
